# Access to surgical assistance: challenges and perspectives^1^

**DOI:** 10.1590/1518-8345.0954.2677

**Published:** 2016-03-28

**Authors:** Maria Fernanda do Prado Tostes, Eduardo Rocha Covre, Carlos Alexandre Molena Fernandes

**Affiliations:** 2 Doctoral student, Escola de Enfermagem de Ribeirão Preto, Universidade de São Paulo, PAHO/WHO Collaborating Centre for Nursing Research Development, Ribeirão Preto, SP, Brazil. Assistant Professor, Universidade Estadual do Paraná, Paranavaí, PR, Brazil; 3 Undergraduate student in Nursing, Universidade Estadual do Paraná, Paranavaí, PR, Brazil. Scholarship holder from Fundação Araucária, Brazil; 4 PhD, Professor, Universidade Estadual de Maringá, Maringá, PR, Brazil

**Keywords:** Health Services Accessibility, Delivery of Health Care, Surgery, Universal Coverage, Nursing, Systems Integration

## Abstract

**Objective:**

to characterize the access to surgical assistance in Brazil.

**Method:**

documentary study, with a quantitative approach, developed from information of the Caixa Preta da Saúde [Health Black Box] database, of the Brazilian Medical Association.

**Results:**

in the one-year period 3773 cases related to health care in Brazil were recorded. There were 458 (12.3%) records on surgical assistance. Of these, most, 339 (74.1%), involved the lack of access in all regions of Brazil. The main access constraint was the prolonged waiting time for surgery. Other constraints were the excessive waiting for medical appointment with experts, doing examinations and cancellation of surgeries.

**Conclusion:**

the access to surgical assistance, by users of the Brazilian health system, is not widely guaranteed, reinforcing the need for integrated governmental actions, organization of the health care network, management of health care and human resources to overcome the challenges imposed to achieve the Universal Access to Health and Universal Health Coverage.

## Introduction

Surgical condition is essential for prevention of chronic disabilities and mortality^(^ 1 ^-^ 2 ^)^. This is because, often, it is the only solution to avoid them in case of injuries, traffic accidents, burns, disasters, violence, obstetrical complications, emergency abdominal and non-abdominal conditions, and others that may significantly affect the quality of life, such as cataract and congenital malformations^(^ 1 ^)^.

Surgery is at the end of the spectrum of the classic curative model. Thus, regardless of successful prevention strategies, surgical conditions will always be responsible for a significant portion of the burden of disease in a population. In developing countries, in particular, where conservative treatment is not readily available, the incidence of trauma and obstetric complications are high and with significant accumulation of untreated surgical diseases^(^ 1 ^)^.

However, the access and coverage of essential surgical care, as part of the human right to health, are not widely guaranteed^(^ 2 ^-^ 3 ^)^. As a result, surgical pathologies are aggravated, affect the socioeconomic condition of the active population, impair the quality of life, and become potentially lethal^(^ 2 ^)^.

In addition to improve the quality of life, the quality of surgical interventions, and increase access to surgery, it can assist in achieving the United Nations Millennium goals for 2015; considering that surgery enhances the reduction of child mortality (goal 4) and improves maternal health (goal 5) for obstetric complications treatment. In addition, surgery may contribute to reduce the number of people living in poverty (goal 1), since surgical conditions can keep people outside the labor market^(^ 4 ^)^.

Thus, considering the importance of the global surgical assistance, its impact on the quality of life of patients, limitation of access and health care coverage, and interest in answering some emerging challenges in nursing, this study aimed to characterize the access to surgical assistance in Brazil.

## Method

Documentary study, with a quantitative approach, carried out from a secondary source of data, of public domain, of the electronic *Caixa Preta da Saúde*[Health Black Box] database.

This database, created in March 12, 2014, is the result of a non-governmental initiative of the Brazilian Medical Association. It is an electronic communication channel with users of health care that aims to receive and compile, into a database, complaints/records on the issues that affect the public and private health in Brazil. Voluntarily, anyone, from anywhere and at any time, can send photos and videos, complaining failures and describing the difficulties faced in the search for health care. Complaints are forwarded to the Public Prosecution for formal verification of the facts reported, thus contributing to the government and managers to take the necessary precautions^(^ 5 ^)^.

We considered as an inclusion criterion the records containing information about surgical assistance in Brazil. To select them, we used the following descriptors: surgery, surgeon, surgery center, to operate, operation, preoperative, postoperative, surgical, anesthesia, and anesthesiologist.

The data were collected in March, 2015. In the electronic database (accessed by the address http://www.caixapretadasaude.org.br) all records were selected for the period of 12 months (March, 2014 to February, 2015), of each Brazilian municipality. After reading each record, we selected those that contained the descriptors previously defined. For the collection, we used a structured instrument, with the following variables: lack of access, waiting time for surgery, waiting time for appointment with a specialist, waiting time for examinations, cancellation of surgery, and surgical specialty.

Statistical analyses were carried out using the Statistica software 12.0. The results were expressed as mean and standard deviation for the quantitative variables and in frequency and percentage for the qualitative variables. We used the Chi-square test to verify differences in the proportions between regions of the country, and the one-way Analysis of Variance (ANOVA) for comparison between average values. For all the analyses, a p<0.05 significance level was considered.

Regarding ethical aspects, we used information from the public domain, present on the Internet. Thus, we obtained an authorization of the responsible people for the database, since they are the trustee of the information. In addition, we followed all ethical principles required for analysis and dissemination of data.

## Results

There are 3773 (100%) records related to health care in Brazil. Of these, 458 (12.3%) involved surgical assistance. Most of the surgical records, 339 (74.1%), involved lack of access in all Brazilian regions, according to Table 1.

**Table 1:** - Distribution of records related to health care regarding the regions, Brazil, 2015

**Region**	Records F (%)*		
	**Healthcare**	**Surgical assistance**	**Lack of access**
Mid-West	220 (5.8)	22 (4.8)	18 (5.3)
Northeast	840 (22.3)	81 (17.7)	64 (18.9)
North	156 (4.1)	16 (3.5)	09 (2.7)
Southeast	2095 (55.5)^†^	267 (58.3)^†^	195 (57.5)^†^
South	462 (12.3)	72 (15.7)	53 (15.6)
Brazil	3773 (100)	458 (100)	339 (100)

*F=Frequency. †Significant difference compared to other regions (Chi-square).

Other records about surgical assistance, 119 (25.9%), comprised hospital care regarding physical structure, such as precarious conditions of hospital facilities and poor hygiene; the given assistance, which included the lack of information about users, humanization and quality regarding the services related to human resources, including medical malpractice and negligence, and also negligence of health professionals with the users and their families (data not shown). We found that the main access constraint was the waiting time for care, at different points of the health care network, with a predominance of waiting list for surgery, according toTable 2. The waiting time for surgical care in Brazil was of 484.74±191.29 days, with regional disparities, according toTable 3.

**Table 2:** - Distribution of constraint factors of access, according to regions, Brazil, 2015

Region F (%)*					
**Access constraints**	**Mid-West (n=18)**	**Northeast (n=64)**	**North(n=09)**	**Southeast (n=195)**	**South(n=53)**
Waiting list for surgery	15 (83.4)	46 (71.8)	07 (77.8)	134 (68.8)	41 (77.3)
Waiting list for appointment with a specialist	05 (16.6)	01 (1.6)	01 (11.1)	12 (6.1)	09 (17.0)
Waiting list for examinations	---	01 (1.6)	---	12 (6.1)	02 (3.8)
Cancellation of surgery	---	16 (25.0)	01 (11.1)	36 (18.5)	01 (1.9)
Failure in the schedule process^†^	---	---	---	01 (0.5)	---

*F=Frequency. †The name of the user was not on the waiting list for surgery anymore.

**Table 3:** - Distribution of mean waiting time (in days) for surgical care, according to region, Brazil, 2015

**Region**	Waiting time (mean±SD)*
Mid-West	332.92±473.23^†^
Northeast	354.48±416.58^†^
North	391.25±426.69^†^
Southeast	494.06±504.17
South	850.97±781.47
Brazil	484.74±191.29

*SD = Standard Deviation. †Significant difference regarding the South region (ANOVA, p<0.05).

## Discussion

Surgical assistance is essential. However, in this study, we noted that, according to*Caixa Preta da Saúde*, the critical center of surgical assistance is predominantly the lack of access of the user to surgery. For users, the prolonged waiting time is the main access constraint to primary health care.

According to the indexed literature, this is the first study that uses information from the *Caixa Preta da Saúde* database. This is a provocative and relevant tool, which gives voice to the Brazilian health system users, and has the potential to constitute an important mechanism of ombudsman. Indeed, it is a tool which exposes the weakness of the public health system in Brazil, illustrating with dramatic tones the difficulty of many people to at least have their complaints heard, and much less met.

On these findings, it is believed that health care in Brazil is often against the fundamental assumptions of Universal Health Coverage, in which all people should have equal access to health actions and services of quality, according to their needs over life. The Universal Health Coverage should be the main objective and guideline of health systems, and its fundamental premise is the right everyone has to the highest standard of health care^(^ 6 ^)^. Moreover, it is a powerful means of promoting health, well-being, and human development^(^ 7 ^)^.

Regarding the number of records, it was observed that the Southeast region significantly surpassed other regions. This greater potential of use of the tool in this region can be due to the higher population density. On the other hand, the Northern region, with smaller number of records, has the lowest population density (3.9 people per km²)^(^ 8 ^)^. Additionally, regarding Internet access in Brazil, there is a marked inequality between regions. Regions with more access, Mid-West, South and Southeast, surpass the North and Northeast regions^(^ 9 ^)^. Thus, this disparity may have limited the use of the tool by Brazilians, particularly in the Northern region.

It is noted that the Southeast, as the main user of the tool, faces limitations to the Universal Health Access and Coverage, similar to other regions, even with higher socioeconomic development and better welfare conditions, regarding the number of beds, specialized hospitals available, and operations per inhabitant when compared to the North and Northeast^(^ 10 ^)^.

Regarding the lack of surgical access, this is not a new problem: health systems worldwide, regardless of the socioeconomic status of the countries, deal with it. In Brazil, the waiting list for elective surgeries is a reality with regional nuances as to procedures with longer or shorter lists, whether measured in number of patients or waiting time^(^ 11 ^)^.

We noted that the waiting comprised all the course of the user in the health care network. Considering that the user, due to the presence of signs and symptoms, accesses the network searching for medical care in the basic care. Once acknowledged the need, there is the referral to the specialist in the outpatient care. After a long process of appointments, research, expert evaluation, and the need for surgical treatment, the user is placed in a waiting list for surgery, which will occur in the hospital care instance^(^ 11 ^)^.

Similarly, a study conducted to verify the barriers to the access to the treatment of cataracts showed a gap between the search for specialized medical care and surgical resolution. The contributing factors were the difficulty in preoperative examinations, number of times the patient needed return to the service (3.2±1.5) as well as the waiting time between the first consultation and the surgery (3.2±2.6 months). The amount spent on the preoperative examinations ranged from 5 to 170 BRL^(^ 12 ^)^.

In this study, we showed an excessive waiting time, over one year, to surgical care in all regions of the country. In the South region, there was a longer waiting time, with significant difference in relation to the Mid-West, North, and Northeast regions. However, studies show variations from 3 to 6-month in waiting time, with more accessible services in the South and Southeast regions^(^ 10 ^,^ 12 ^)^. It is recognized that the incorporation of records is voluntary and depends on the users with access to Internet. Thus, we should be cautious in interpreting this result, because official data from health services can scale more precisely the reality of the waiting list for the service in the regions of the country. Despite this constraint, this result shows a cruel and legitimate reality, complained by users who were drastically underserved.

It should be noted that the prolonged waiting for care occurs when demand is greater than the capacity of the public system. Often, the list reflects the inadequacy of public funding for health. And is one of the main problems of public health systems, being a permanent source of political and social discontent^(^ 13 ^)^.

Achieve Universal Access to Health and Universal Health Coverage is a permanent, complex and challenging task^(^ 14 ^)^. Data from *Caixa Preta da Saúde* show that, in addition to the predominance of lack of access, in certain situations, the prolonged waiting time on the list for surgery does not ensure the care. Eventually, the user deals with a new obstacle, the cancellation of surgery as a result of inadequate infrastructure. In addition, when users access the hospital care, the infrastructure is precarious, and there is no quality in the assistance offered.

In Brazil, despite the urgent need for tackling the current challenges relating to achieve Universal Access to Health and Universal Health Coverage, it should be recognized that, in health, an advance landmark was the health reform, which culminated in the creation of the Unified Health System (SUS). The result of the struggle of Brazilian society, politics and government actions, provided a significant expansion of access to health care, through the Family Health Strategy^(^ 7 ^)^.

Following this direction, from 2014, member states of the World Health Organization/Pan American Health Organization have been focusing on concurrent and independent actions to expand the equitable access to complete health services, of quality, and focusing on the people and communities, to promote the expansion of Universal Access to Health and Universal Health Coverage^(^ 6 ^)^.

More specifically on access to surgery, we highlight the mutual-aid group of surgeries, government-funded, through allocation of extra resources. The mutual-aid group aims to meet the repressed surgical demands through concentration of efforts of the health services to accomplish a significant number of surgeries in a short time^(^ 11 ^)^. However, what was supposed to be an emergency strategy has become the *modus operandi* for access, being a palliative measure, costly to the system and that does not guarantee the access to surgical assistance in a sustainable way, since the extended waiting time in the list for surgery persists, with negative impact on health and quality of life of patients^(^ 11 ^)^.

In relation to the political and economic aspects, constraints to the access to health in Brazil, we noted that the public investment in health system remains, unacceptably, below the desired levels and, significantly, lower than international standards. Thus, the public spending on health care was responsible for only 48% of total health expenditure, accounting for only 7.2% of the total public expenditure, significantly less than the total value of 14.3%. The total expenditure on health per capita per year is, on average, 427 USD. From this amount, only 204 USD were invested by the government, a much lower value when compared to an overall expenditure of 429 USD per capita or for investment from developed countries such as the United States of America (3,076 USD per capita) or Europe (1,350 USD per capita)^(^ 3 ^)^. This ensures a gap favorable to the offer of a private health system, which firmly established in a neoliberal policy guarantees the access to who can afford it, increased the fact that, at the highest level of power, it is used as lobby and political influence to perpetuate a cycle of abandonment of the public health system and favoring the private initiative. In summary, it is said that the State deserts its function as provider of the citizens' health, to deliver it as a consumer or customer to private agents.

When it comes to surgery, there are public investment restrictions similar to those mentioned above, confirming the finding of a government practice of severe public health under-funding. From 1995 to 2007, 32,659.513 non-cardiac surgeries were analyzed. There was substantial increment of 20.42% in the number of surgical procedures. However, the epidemiology of surgeries in Brazil differs considerably, from international standards. Annually, about 38 million surgeries are held in the United States of America, and 7 million in Europe. In Brazil, there were approximately 3 million non-cardiac surgeries. Additionally, it was noted that the costs of the procedures are lower, and the mortality rate is higher in developed countries. Thus, we concluded that the access to surgical interventions is poor and uneven, with insufficient number of surgeries accomplished, and surgical results are even worse when compared to international standards^(^ 3 ^)^.

Therefore, only a systemic change is able to overcome the challenges imposed to achieve the Universal Access to Health and Universal Health Coverage^(^ 14 ^-^ 15 ^)^. Thus, it is believed that an integrated approach, which includes endless joint efforts in the political and economic aspects, in the organization of the health care network, management of health services and human resources, will enable to overcome these challenges, in such a way that health and other social rights of Brazilian citizens are widely guaranteed.

Regarding the political aspect, it is imperative that government policies face the challenges related to the macroestructure complexity, so that they can overcome the challenges imposed to health. Thus, for improving health, it is necessary to improve governance, i.e., a greater degree of responsibility of the public sector, less corruption, and assumption that provide the public health system is a constitutional duty of the State^(^ 7 ^,^ 14 ^)^. Furthermore, given the social determination of health, the economic crisis and social inequality must confront, education must be improved, the environment must be preserved, and the increasing demands of an ageing population must be met^(^ 14 ^)^. Equally important, the influence of the medical and pharmaceutical industry in the health sector must be mitigated, which is often subservient to the interests of the market, to the detriment of the interests of the population, among others.

Regarding the health care network, to optimize the deployment of mechanisms and strategies of organization and integration of assistance network contributes to promote the capacity of health systems to provide more coordinated care, minimize access barriers between assistance levels, and a health care more synchronized and in a proper time. However, it should be recognized that one of the main obstacles to the integration of the health services network is the prolonged waiting time for the care^(^ 16 ^)^.

Regarding the organization of the network to surgical assistance, aiming to improve the access and qualification of social assistance practices, the access to specialized and hospital services should occur, predominantly, through reference of Family Health Strategy. Additional actions include the development of protocols to facilitate the access to specialized and hospital care. To undertake efforts along with other professionals, to further integration, exchange and information continuity between basic and specialized care. Definition of access measures, such as the waiting time for primary care, consultations with specialists, diagnostic tests, and waiting for surgery and, based on these indicators, to establish strategies for better management of waiting^(^ 16 ^-^ 17 ^)^.

In outpatient level, we recommend instituting careful research about the needs of the users in a waiting list situation, namely: clinical criteria such as health status deterioration, disease evolution, disabling pain, and mobility limitations; socioeconomic criteria concerning the ability to live and work independently, in addition to the emotional aspects. This allows the user the re-evaluation of priority while in the waiting list, and reconducting the family health team, for continuous monitoring^(^ 13 ^,^ 17 ^)^.

In addition, encouraging the development of guidelines for determining safe and acceptable waiting period, development of prioritization tools of assistance to consider the psychosocial characteristics, definition of responsibilities to operationalize the prioritization process, being a surgeon, nurse or other health professional^(17).^

Strictly regarding to the management of resources, comparisons show that greater supply of beds, health professionals and health expenses are effective strategies, in long-term, in management of the waiting. Thus, the ability of the health services can be optimized. We suggest the payment for productivity, quantitative and qualitative investment in human resources, evidence-based practice support, and use of idle capacity of surgical center on weekends, among others^(^ 13 ^,^ 17 ^)^.

Human resources are considered one of the central pillars for Universal Access to Health and Universal Health Coverage^(^ 18 ^)^. Especially nursing, due to the constraint potential in completely meeting the needs of the population, in the different points of the health care network. However, deep imbalances and gaps remain in the availability, distribution, composition, competence and productivity of health human resources, especially in primary health care^(^ 18 ^)^.

Thus, enhancing the scope of nursing practice requires political will for change. Notably, in the political field, it is essential the development of infrastructure policies that support the nursing profession, including regulation, scope of practice, certification, undergraduate studies, graduate studies, continuing education and wage reform^(^ 15 ^)^.

In the field of professional performance, nursing, in partnership with other health professions, must assume the commitment to public health, through the construction and consolidation of the SUS. To make this commitment involves overcoming Cartesian health assistance practices, aiming the completeness of the care, introjecting the premises of health policy in force, incorporate them into your practice, and act in the organization of health care networks, with emphasis on primary health care.

In the field of training, nursing must have the commitment to the construction of values that structure it as a social practice, provided through the interpretative looks of its history and open to the current reality, considering the political, economic and social structure in force in the country, health care models and their coverage, as well as the internal struggles of workers in the process of work and future challenges^(^ 19 ^)^.

## Conclusions

According to the *Caixa Preta da Saúde* database, most records about surgical assistance involved the lack of access in all regions of Brazil. The main access constraint was the prolonged waiting time for surgery. Thus, we observed that the access to surgical assistance, by the Brazilian health system users, is not widely guaranteed. This reinforces the need for integrated governmental actions, organization of the health care network, management of health care and human resources to overcome the challenges imposed to achieve the Universal Access to Health and Universal Health Coverage.

Therefore, if Universal Health Coverage is assumed as a government priority and also of health services, it will stimulate the nursing to expand its role in the construction of a system of universal, equitable and complete public health.
